# Characterization of a set of novel meiotically-active promoters in *Arabidopsis*

**DOI:** 10.1186/1471-2229-12-104

**Published:** 2012-07-09

**Authors:** Junhua Li, Andrew D Farmer, Ingrid E Lindquist, Stefanie Dukowic-Schulze, Joann Mudge, Tao Li, Ernest F Retzel, Changbin Chen

**Affiliations:** 1 Department of Horticultural Science, University of Minnesota, 1970 Folwell Avenue, St. Paul, MN, 55108, USA; 2 National Center for Genome Resources, 2935 Rodeo Park Drive E, Santa Fe, NM, 87505, USA; 3 College of Life Sciences, Henan Normal University, Xinxiang, Henan, 453007, China

**Keywords:** Meiosis, Homologous recombination, Promoter, GFP, *cis*-regulatory elements, Plant molecular breeding

## Abstract

**Background:**

Homologous recombination, together with selection, laid the foundation for traditional plant breeding. The recombination process that takes place during meiotic cell division is crucial for the creation of novel variations of highly desired traits by breeders. Gaining control over this process is important for molecular breeding to achieve more precise, large-scale and quicker plant improvement. As conventional ubiquitous promoters are neither tissue-specific nor efficient in driving gene expression in meiocytes, promoters with high meiotic activities are potential candidates for manipulating the recombination process. So far, only a few meiotically-active promoters have been reported. Recently developed techniques to profile the transcriptome landscape of isolated meiocytes provided the means to discover promoters from genes that are actively expressed in meiosis.

**Results:**

In a screen for meiotically-active promoters, we examined ten promoter sequences that are associated with novel meiotic candidate genes. Each promoter was tested by expressing a GFP reporter gene in *Arabidopsis*. Characterization of regulatory regions revealed that these meiotically-active promoters possessed conserved motifs and motif arrangement. Some of the promoters unite optimal properties which are invaluable for meiosis-directed studies such as delivering specific gene expression in early meiosis I and/or meiosis II. Furthermore, the examination of homologs of the corresponding genes within green plants points to a great potential of applying the information from *Arabidopsis* to other species, especially crop plants.

**Conclusions:**

We identified ten novel meiotically-active promoters; which, along with their homologs, are prime candidates to specifically drive gene expression during meiosis in plants and can thus provide important tools for meiosis study and crop breeding.

## Background

Meiosis is a key feature in the life cycle of flowering plants during which homologous chromosome pairing, synapsis and recombination are achieved [1-3]. Understanding the mechanisms of the meiotic process is crucial for not only the cell cycle regulation, but also plant breeding, because homologous recombination ensures genetic exchange between homologous chromosomes, generates the genetic variations, and maintains the inheritance of traits. Genome-wide gene expression analyses on isolated meiocytes revealed unique transcriptome-landscapes during male meiosis in the model systems of *Arabidopsis* and rice [4-7]. Over 1,000 protein coding genes demonstrated preferentially expression in male meiocytes, with a group of 55 genes that have mitochondrial genome origins, and 1,036 transposable element genes were up-regulated in male meiocytes [5]. The observation suggested that there is likely a specific transcription-regulatory mechanism during meiosis. As the first step toward understanding the molecular mechanism, we focus on characterizing the function and regulatory elements in selected candidate meiosis-gene promoters of this study. The objectives are to find common regulatory features in meiotically-active promoters and to explore the potential for applying the promoters in plant meiosis studies and crop breeding.

A prerequisite for meiotical engineering is the availability of effective meiotically-active promoters. However, the widely used CaMV 35 S promoter is not efficient in meiocytes. For example, *AtCDC45* encodes a protein required for the normal fertility of the model plant *Arabidopsis,* and when an *AtCDC45*-RNAi construct driven by the 35 S promoter was transformed into wild type, only 20 of 59 transformants became sterile (34%), whereas a greater percentage of sterile plants (61%, 45 of 74 transformants) could be obtained by replacing the 35 S promoter with the meiosis-specific *DMC1* promoter [8].

So far, only a limited number of meiotically-active promoters has been reported and investigated. The expression of the meiotic recombination gene *AtDMC1* has been reported to be restricted to meiotic cells in anthers and carpels, and a β-glucuronidase (*GUS)* reporter fused to an *AtDMC1* promoter revealed that the reporter gene activity initiated at the stages where meiosis takes place [9]. However, activity of the *AtDMC1* promoter is not restricted to meiotic cells [9-11]. *MS5* is a gene essential for male meiosis [12]; *in situ* hybridization showed that *MS5* is localized specifically within anther cells undergoing meiosis [13].

In yeasts, rodents and human, the expression of genes in meiosis has been well studied [14-18]. The male meiocytes of *Arabidopsis* are of an extraordinary small size (1% of anther tissues) and are surrounded by somatic anther lobes, making the isolation and analysis of *Arabidopsis* meiocytes challenging. Recently, the application of effective meiocyte collection methods made it possible to investigate the meiotic transcriptome profile [5,6], thus allowing the bulk isolation and characterization of meiotically-active promoters. In this study, we experimentally verified the activity of twelve meiotically-active promoters out of fifteen candidate promoters, including ten new promoters.

Transcriptional regulation is critical for many developmental processes, making it important to analyze the transcriptional control to better understand the mechanisms that control spatial and temporal patterning in development [19]. The bulk isolation and characterization of meiotically-active promoters makes the study of important novel *cis*-regulatory motifs in these sequences feasible.

Comparative transcriptome analysis revealed similarity in meiocyte transcriptomes between organisms, for example, more than 500 single-copy genes are shared by meiotic cells of *Arabidopsis*[6], mouse (*Mus musculus*) [14] and fission yeast (*Schizosaccharomyces pombe*) [18], with a larger number of genes expressed in both mouse and fission yeast, or *Arabidopsis* and mouse. Therefore, analyzing meiotically-active promoters from *Arabidopsis* should provide not only clues of meiotic regulatory networks in *Arabidopsis*, but can also give hints for other species. These meiotically-active promoters or their close relatives could evolve into substantial tools for molecular breeding across species, especially in the plant kingdom where crop improvement is highly desired to cope with global climate changes and the increasing world population.

## Results

### Identification of meiotically-active promoters

**Figure 1:**
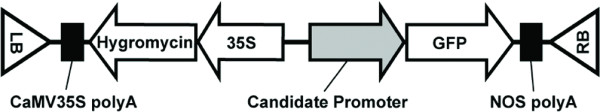
**Schematic representation of the expression modules in pCAMBIA1302.** CaMV35S polyA: Cauliflower mosaic virus 35 S poly-A terminator; 35 S: Cauliflower mosaic virus 35 S promoter, NOS polyA: Nopaline synthase poly-A terminator; GFP: Green fluorescent protein; Hygromycin represents the plant resistance selectable marker. LB: left border of the T-DNA, RB: right border.

A previous study performed in our labs compared the transcriptomes of whole anthers, isolated meiocytes and seedlings of *Arabidopsis thaliana*[5]. Among the transcripts that were found to have twofold or greater changes between both meiocytes versus seedlings and anther versus seedlings, we chose 15 candidates to further study their promoters (Table 1). These include two promoters of the previously reported meiosis-specific genes, *AtDMC1*[9,10] and *MS5*[12,13], which serve as positive controls, and were confirmed in their meiosis-preferential expression were also confirmed in our profiling study [5]. Since the 5' UTR (untranslated region) may have roles in the regulation of gene expression [20], all cloned promoters end one nucleotide ahead of the start codon of the annotated coding region. To generate a reporter system, putative promoters were amplified and cloned into the binary vector pCAMBIA1302, just upstream of the green fluorescent protein (GFP) coding sequence (Figure 1). A promoter which shows similar activity as the control promoters, marked by GFP fluorescence, is defined as a meiotically-active promoter. In addition, we checked for GFP expression in somatic cells of roots, stems and leaves of adult plants to exclude promoter activity during the major vegetative growth.

**Table 1:** A List of gene IDs and associated primers for the 15 analyzed promoters

**Promoter name**	**Primer information**	
	**No.**	**Sequences**
(pATDMC1) * pAT3G22880	OMC1657	GCGTCGACTGGTAGAGTCATGTTACTTAAGGT
	OMC1656	GGACTAGTCCATGGTCTCGCTCTAAGAGTCTCTAA
pAT3G19070*	OCC555	ACGCGTCGACTCTTCAACATCAACCCGACC
	OCC556	CATGCCATGGCTTGCAACTTAAGAAATTTGATTC
pAT2G28090*	OCC563	ACGCGTCGACTGGAAACTTAATGCAAACGC
	OCC564	CATGCCATGGTCGTCTCTAACTTCTTCTGC
(pMS5) * pAT4G20900	OCC553	ACGCGTCGACCTCGGCAAACGCCATAAC

	OCC554	CATGCCATGGTCTTTTTCGATTCTCTCTGTC
pAT1G26510*	OCC557	ACGCGTCGACTTATTGCTCCCAACACTCG
	OCC558	CATGCCATGGTCAATCGCTCTTGTTTCG
pAT2G32310	OCC561	ACGCGTCGACTACTTGGGTGCTTTCTTGTG
	OCC562	CATGCCATGGTAACTTCTTTCCAAAGAATCTC
pAT3G49830*	OCC565	ACGCGTCGACTAAGACTGATTTGCCAACAAGG
	OCC566	CATGCCATGGTCGGTTGTTGAGTTCACC
pAT4G40020*	OCC567	ACGCGTCGACGGGGTTTAGGTCTTTCCAT
	OCC568	CATGCCATGGATGATAGAATGTTTTTTATTCAGC
pAT2G21640*	OCC569	ACGCGTCGACTGAAAGGTTTCCCACTCC
	OCC570	CATGCCATGGGAAAACAGAAAGAAATCTCATG
pAT3G07250*	OCC571	AACTGCAGGCTTCGCAAATCCAACCT
	OCC572	CATGCCATGGATAAAGATTCAACAAACATATAATGTC
pAT2G31141*	OCC573	ACGCGTCGACCCATACAGAGTAAGCCAAACC
	OCC574	CATGCCATGGCCCTCCGACTTTAGAATCC
pAT1G15320*	OCC575	ACGCGTCGACCAACTCACCACCTCCCTCT
	OCC576	CATGCCATGGTCGTGTTTCTTCTTCAGCACT
pAT1G64625*	OCC577	ACGCGTCGACTTTCCTTGCTTGTGATCTTC
	OCC552	CATGCCATGGTTAGGAATCCAAGCCGGG
pAT3G52770	OCC648	ACGCGTCGACTACAAAATGGTCCAAAACGG
	OCC649	CATGCCATGGCTGCTTTCTTGCTACAAGTAAAAC
pAT1G24220	OCC625	ACGCGTCGACGAAGACATGAGATTTTGGGGTCA
	OCC560	CATGCCATGGCTAAACCCTCCAAG

* refers to meiotically-active promoter. For each primer pair, the former one is the sense primer, the later one is the antisense primer; The *Pst*I (CTGCAG) or *Sal*I (GTCGAC) sites in sense primers, the *Nco*I (CCATGG) sites in antisense primers are underlined.

**Figure 2:**
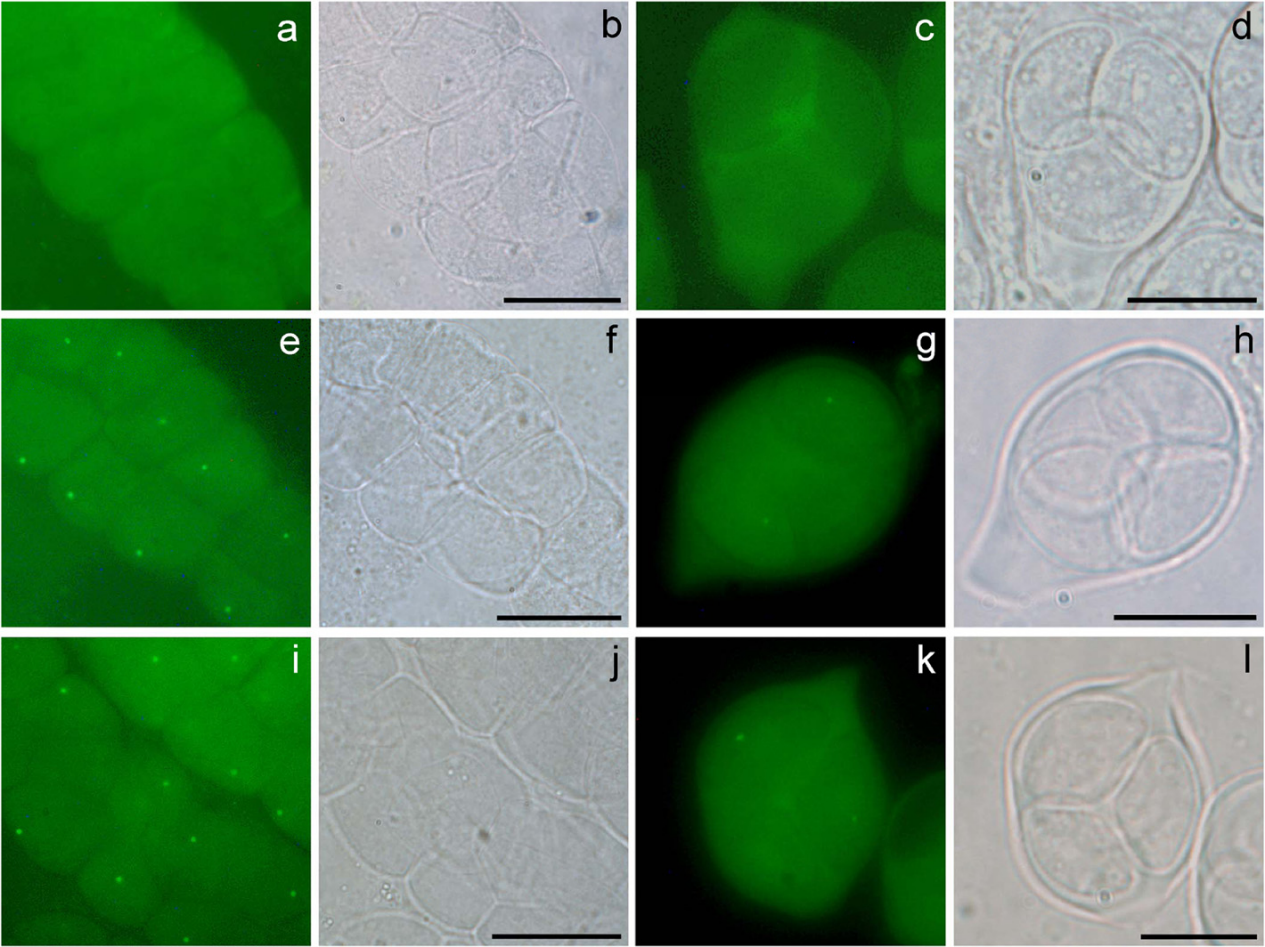
**GFP signals in meiocytes driven by meiotically-active promoters.** (**a**-**d**), wild type control meiocytes, a and c showing the normal weak background fluorescence; (**e**-**h**), *pMS5:GFP* meiocytes as a positive control, e and g showing GFP signals; (**i**-**l**) *pAT3G19070:GFP* meiocytes, i and k showing similar GFP signals as the positive control. (**a**, **e**, and **i**), early meiosis I cell clusters; (**c**, **g** and **k**), meiosis II meiocytes; images on their right are corresponding bright-field images, respectively. Scale bars, 10 μm.

Analysis of transgenic lines containing the GFP reporter driven by a candidate promoter showed that out of fifteen candidates, twelve expression modules had detectable GFP fluorescence within male meiocytes (Table 1, Figure 2 and Additional file 1, Figure S1). These include the expression modules containing the promoters of *AtDMC1* and *MS5* (Table 1), supporting the reliability of the GFP reporter system. In addition to these two already known promoters, ten additional meiotically-active promoters were newly identified in this study (Table 1, Figure 2 and Additional file 1, Figure S1). The particular expression pattern of the GFP modules were common to all positive lines, showing a single small and perinuclear spot per cell (Figure 2Additional file 1, Figure S1 and Additional file 2, Figure S2). In addition, we observed GFP signal in both nuclei and cytoplasm of *pAtDMC1:AtDMC1:GFP* meiocytes, which is similar to the CYCA1;2-GFP fusion [21]. In *pAtDMC1:GFP* meiocytes, however, the GFP signal was only detected in the cytoplasm and as a perinuclear green spot (data not shown), which is similar to all other tested promoter-GFP fusions in this study. No GFP signal was observed in somatic cells, such as anther walls ( Additional file 2, Figure S2). There are two visible types of meiocytes within an anther after dissection: I, cells that form columns (Figure 2a, b, e, f, I and j) contain meiocytes undergoing the prophase stages of meiosis-I; II, dissociated meiocytes (Figure 2c, d, g, h, k and l) which include meiosis-II cells. Most meiotically-active expression modules yield GFP signals in both cell types, thus spanning the whole duration of meiosis-I and II. However, module *pAT1G15320:GFP* only resulted in a signal in dissociated meiocytes ( Additional file 1, Figure S1), indicating expression restricted to later meiosis, whereas module *pAT4G40020:GFP* gives detectable signals only in cell columns ( Additional file 1, Figure S1), pointing to an early meiosis preferential expression pattern. For the ten examined promoters, no specific fluorescence signal was observed in other examined tissues, such as roots, stems and leaves of adult plants (data not shown), adding evidence to the meiotic specificity of these promoters, and the GFP signal was also not observed in meiocytes in the wild-type control (Figure 2a-d).

In control tests, the levels of fluorescence driven by 35 S promoters were determined, including a multicolored set of organelle markers of endoplasmic reticulum, Golgi apparatus, tonoplast, peroxisomes, mitochondria and plastids [22]. The results showed that the 35 S promoter does not lead to the expression of a detectable level of fluorescence in meiotic cell columns, tetrads and pollen. As an example, Additional file 3, Figure S3 shows a signal from an endoplasmic reticulum marker [22] under the control of a 35 S promoter with dual enhancer elements (d35S) in somatic cells while being undetectable in meiotic cells.

### Enriched regulatory elements in meiotically-active promoters

Gene expression is often regulated by the interaction of transcription factors and target *cis*-regulatory DNA elements in promoters. The identification of potential regulatory elements acting in meiotically-active promoters can be a useful tool for understanding regulatory networks [e.g. [20,23-26]]. We scanned enriched *cis*-acting regulatory DNA elements (CREs) in the promoters of our study to obtain clues about possible co-regulation of meiotically-active genes.

**Figure 3:**
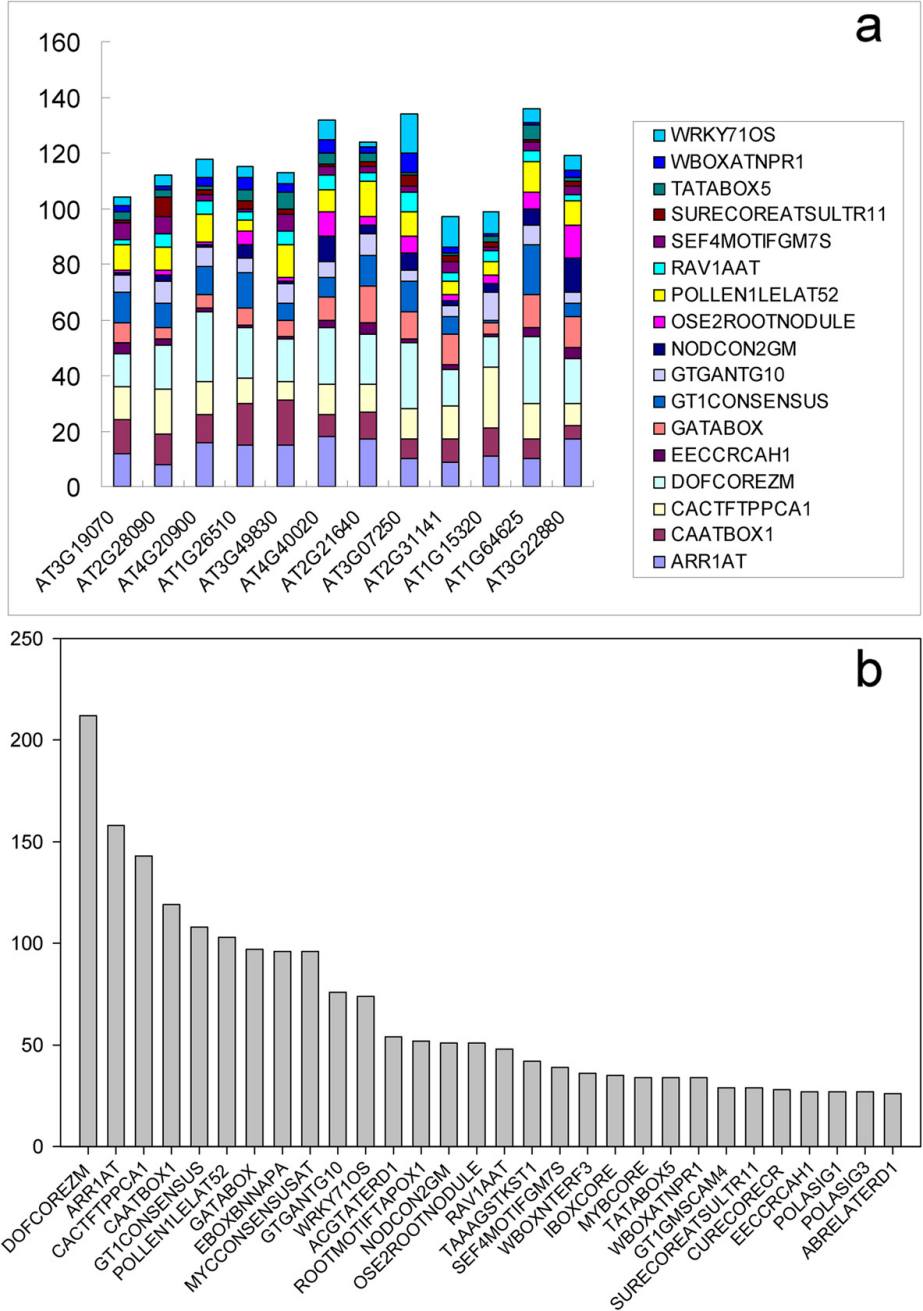
**Enriched*cis*-acting regulatory DNA elements in meiotically-active promoters.** (**a**), occurrence of 17 common CREs that distributed in all 12 promoters; (**b**), occurrence of top 30 most abundant CREs with a total occurrence above 26, or an average occurrence above two per promoter. See Additional file 4, Table S1 for description of each motif.

We first analyzed known plant regulatory elements in these promoter sequences by using the PLACE collection. We considered CREs within one kb upstream of these genes, since the effective regions of individual promoters are still unknown, and the normalized length also facilitated a better comparison between different sequences. The names of 17 broadly distributed CREs which appeared in all 12 promoters, and their occurrence in each promoter are shown in Figure 3a and Additional file 4, Table S1. These 17 CREs also rank in the 30 top most abundant CREs (with a total occurrence above 26, or an average occurrence above two per promoter) (Figure 3b, Additional file 4, Table S1).

Several enriched PLACE motifs are universal or structural CREs that seem also common in meiotically-active promoters, such as TATABOX5 [27], POLASIG1 and POLASIG3 [28-30]. Interestingly, ROOTMOTIFTAPOX1 [31], NODCON2GM [32], RAV1AAT [33], OSE2ROOTNODULE [34,35] are all consensus CREs in root and nodule, pointing to a common property of these cells and meiocytes, likely due to their being either in the mitotic or the meiotic process. Many CREs are environment responsive motifs, for example, MYCCONSENSUSAT for cold [36-38], WRKY71OS for gibberellin and pathogenesis [39,40], ACGTATERD1 and IBOXCORE for light [41,42], MYBCORE for water stress [43], GT1GMSCAM4 for pathogen and salt [44] and WBOXNTERF3 for wounding [45]. Additionally, there is a high similarity to motifs in the promoters of rice sperm-cell-specific genes: the examined meiotically-active promoters share 9 out of 10 common motifs associated with rice sperm cell-specific genes, whereas one detected motif (ROOTMOTIFTAPOX1, ATATT) was only shown in sperm cell-specific genes [46].

**Figure 4:**
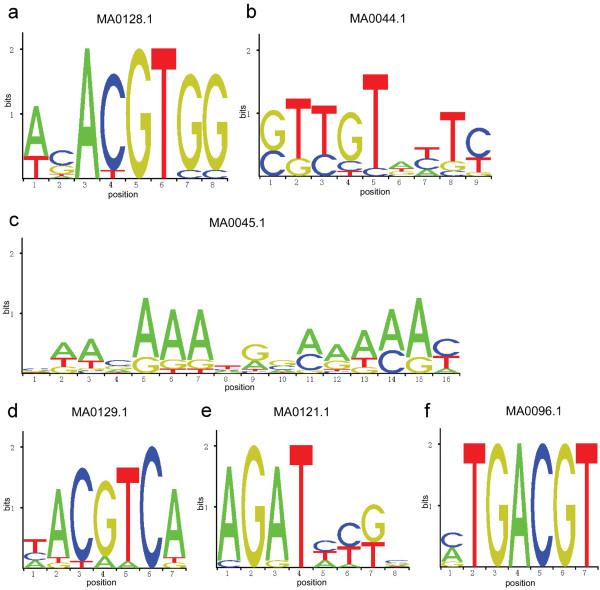
**Sequence logos of overrepresented sequences in meiotically active promoters, found by Pscan.** Letters abbreviating the nucleotides (A,C,G,T) in the images are sized relative to their occurrence. See Additional file 5, Table S2 for description of each motif.

To complement searches for possible motifs that have a statistically overrepresented frequency in the genome, we computationally analyzed the twelve promoters using Pscan [47]. As a result, six motifs were found enriched within the twelve promoters (*p* < 0.1, Figure 4 and Additional file 5, Table S2).

Among these motifs (Figure 4 and Additional file 5, Table S2), MA0044.1 (*p* = 1.52751e-02) and MA0045.1 (*p* = 4.32294e-02) are binding sites for the chromatin-associated proteins HMG-1 and HMG-I/Y [48]. The high-mobility group proteins (HMG) are a group of chromosomal proteins that help with transcription, replication, recombination and DNA repair [49]. MA121.1 (*p* = 9.30809e-02) is a binding site of ARR10, whose multifunctional domain is responsible for both nuclear localization and DNA binding [50]; MA0096.1 (*p* = 9.96644e-02) is a binding site of two flower-specific bZIP proteins [51]. These motifs are likely basic elements that confer tissue- or developmental stage-specific activities to their promoters. Additionally, the motifs MA0128.1 (*p* = 9.03657e-03) and MA0129.1 (*p* = 4.67192e-02), which were implicated in abscisic acid (ABA)-mediated stress and light signaling [52], respectively, are found enriched in these promoters ( Additional file 5, Table S2), consistent with the notion that the meiotic process is sensitive to environmental factors and exogenous hormones including light and ABA [e.g. [52-55]].

Furthermore, we used MEME software to search for possible unknown CREs [56]. Three consensus motifs were found present in these promoter sequences ( Additional file 6, Figure S4). These motifs are characterized by enrichment of adenine (or thymine in the reverse complement strands) ( Additional file 6, Figure S4). Similar results were obtained with MClip tool [57] (data not shown). These adenine-rich motifs could be specific binding sites of transcriptional factors and enhancers [58]. Interestingly, the adenine-rich motifs were also found in promoter regions of 15 selected genes with a documented function in meiosis ( Additional file 7, Table S3 and Additional file 8, Figure S5).

### Homologs of the examined meiotically-active genes

**Figure 5:**
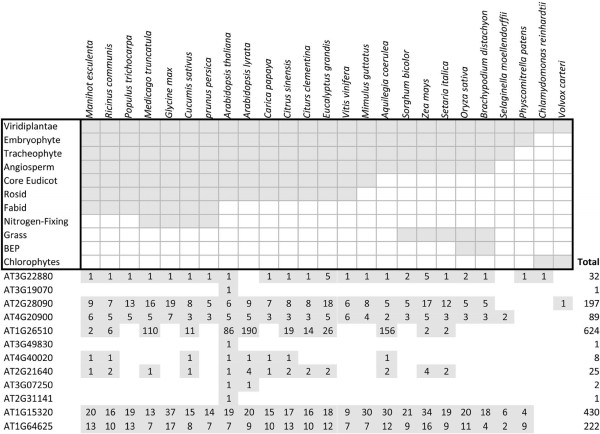
**Examination of putative homologs of meiotically-active genes in green plants.** The upper panel displays the evolutionary clades represented by complete genome sequences in the Phytozome database. The evolutionary distance is shown on the left of the upper panel, with Viridiplantae representing all plants, and extending down into deeper evolutionary distances. For example, *Arabidopsis* is part of the Rosid clade and maize (*Zea mays*) is part of the Grass clade. The individual genes whose promoters were examined are shown in the lower panel, and the grey shading in cells indicates the breadth of their representation in plants. The number of genes similar to the gene whose promoter was characterized in that plant species is shown by the number in the box.

To know whether these meiotically-active genes have homologous sequences in other plant species, especially in crops, we investigated the “family history” of the 12 genes whose promoters were characterized in this study. As is shown in Figure 5, AT3G19070, AT3G49830 and AT2G3114 seem to be specific to *Arabidopsis thaliana* alone while AT3G07250 has at least a putative homolog in *Arabidopsis lyrata*. The other 8 genes are broadly distributed in different taxa, mostly found in flowering plants (Angiosperm), including many important crop species such as soybean (*Glycine max*), maize (*Zea mays*), rice (*Oryza sativa*) and Sorghum (*Sorghum bicolor*). Therefore, we assume that for these genes, there is a higher possibility that similar regulatory networks may be shared among different flowering plants.

## Discussion

The objectives of this study were to identify functional promoters that drive gene expression in meiosis and to find the common *cis*-regulatory elements that are present in all promoters. Results for meiosis-I, during which homologous recombination occurs, are of special interest to predict homologous recombination-related promoters in crop plants. We have tested 15 promoters that are associated with candidate meiosis genes that were discovered by a previous RNA-Seq experiment on isolated meiocytes [5], which include 13 promoters of functionally unknown genes and two reported meiotic gene promoters (pDMC1 and pMS5). Among the 13 candidate promoters that have no documented function in meiosis, ten have shown meiotic activity (≈77%) by driving the expression of GFP signal in meiocytes, thus revealing that our preliminary data has a high reliability for isolating meiotically-active promoters (Table 1). No GFP signal was observed in three transgenic lines of the candidate promoters (Table 1), although their respective gene transcripts were up-regulated in meiocytes in the RNA-Seq study. This may be attributed to distinctions in developmental age, time of harvest, sensitivity of the used method, the chosen promoter region or that these promoters function in a chromosome positional dependent manner [59,60]. Nevertheless, our work provided a significant number of promoters that can drive gene expression in meiosis. These promoters could evolve to be invaluable tools to drive meiotically-active expression in further fundamental meiosis studies as well as in applied molecular breeding.

Until now, most researchers use ubiquitous promoters such as the CaMV 35 S promoter to over-express genes in plants for functional analysis [61]. Those “ubiquitous” promoters, however, are inadequate for meiotic purposes, because they drive gene expression in meiosis at an insufficient level [62]. For example, fluorescent organelle markers [22] that are driven by a 35 S promoter demonstrated no signal in meiosis stages ( Additional file 3, Figure S3), although there is a strong GFP signal detected in somatic cells ( Additional file 3, Figure S3). In accordance with that, RNAi knock-down of a meiotic mutant is also achieved better by using the meiosis-specific *DMC1* promoter than by using the 35 S promoter [61% vs 34%, [8]]. However, even the established and broadly used *DMC1* promoter has its disadvantages in meiosis studies: Doutriaux et al. reported that *AtDMC1* is expressed in mitotically active cells from a suspension culture and is even regulated during the mitotic cell cycle, linking it to the processes in proliferating cells [10]. Furthermore, the *AtDMC1* promoter has been used for studies in young seedlings, yielding an efficient expression in a recombination reporter system [11], which is in accordance with the expression data for *AtDMC1* obtained with ATH1 microarray chips: The eFP Browser tool displays that *DMC1* is also highly expressed in vegetative rosette leaves and especially in the shoot apex and seedling roots [63]. Thus, there is no ultimately optimal meiosis-specific promoter in broad use yet.

The novel candidate meiotically-active promoters from this study should provide more powerful tools for a strict or specified meiotic expression. In our experimental setup, we first chose genes that are highly expressed in meiocytes [5]. We then relied not only on the positive expression that we got with our GFP reporter in meiotic cells but also looked at other tissues, e.g. roots, leaves and stems to validate its nonexistence there. Therefore we defined the promoters here as “meiotically-active” or “homologous recombination-related”, although we cannot completely exclude promoter activity in specific developmental stages or special conditions not covered or detectable by our setup. The decision of which promoter might be best depends on the special application and the preferences of the user, for example if a low or high expression is desired or if the expression should be restricted to a very specific time point in meiosis.

Interestingly, we observed diversified expression patterns in different cell types of meiocytes (cell columns and dissociated meiocytes) resulting from the examined promoters (Table 1). Transgenic lines harboring *pAT1G15320:GFP* only showed a specific fluorescence signal in dissociated meiocytes but not in meiocyte cell columns ( Additional file 1, Figure S1), which suggested a preferential activity in meiosis-II or after homologous recombination. In contrast, *pAT4G40020:GFP* plants showed detectable GFP signals only in early meiosis-I meiocytes, pointing to a homologous recombination-specific promoter. In addition to our results that pAT4G40020 drives gene expression at a high level during early meiosis, microarray data of developmental stages indicates that At4G40020 is further expressed only in microspores [eFP Browser, [63]]. There is also microarray data available for some of our other candidate genes, but not for all of them. Thus, we can confirm their meiosis-specific expression with our experimental setup but cannot completely rule out expression outside meiosis under special conditions or in specific developmental stages. Taken together, we have identified and validated 12 meiotically-active promoters and two of these promoters can be used to specifically address questions regarding roughly meiosis-I (*pAT4G40020*) or meiosis-II (*pAT1G15320)*. For molecular engineering, expressing genes during prophase-I, the stage of recombination, will be of utmost interest.

Given the complexity and a relatively long duration of meiosis (for example, prophase I lasts 21.3 h) [64], the temporal specificity of different promoters might be even more confined to individual meiotic stages. In future work, it will be important to test this possibility and investigate the expression even closer to obtain stage-specific promoters which are powerful tools to meet different requirements.

The confirmation of the meiotic activity of the examined promoters also points to a meiotic function of the respective genes. In addition to the already characterized genes *AtDMC1* and *MS5*[9,12,65,66], we have discovered an additional key gene with a role during meiosis by checking the T-DNA insertion mutants for the ten genes without documented function (unpublished data).

The gene transcription in eukaryotes is complex and is largely modulated by transcription factors that bind to regulatory elements within promoters. We scanned the identified promoter set for motifs with binding specificity for known transcription factors from the PLACE collection (Figure 3 and Additional file 4, Table S1) and used the software tool Pscan (Figure 4 and Additional file 5, Table S2). CREs that are common to the meiotically-active promoters from this study may reflect common binding sites for certain transcription factors that are required for meiotic activities (such as the binding sites of HMG-1, HMG-I/Y, ARR10 and bZIP910, Additional file 5, Table S2). It also provides a hint as to know how these promoters are shared by stimulus–response pathways (such as the binding sites of EMBP-1 and TGA1A, Additional file 5, Table S2). We also analyzed the promoters of 15 genes with a documented function in meiosis ( Additional file 7, Table S3) with Pscan. Although they are not all meiosis-specific under the criteria used in [5] Chen et al. (2010), the identified common elements include not only “basic element” such as HMG-1 binding sites, but also binding sites for proteins involved in gibberellin response and leaf development ( Additional file 9, Table S4). Therefore, it appears that the crosstalk between meiosis and environmental signals, especially hormone signals, are largely through their promoters. These identified CREs can also be further used to design the experimental verification of regulatory elements and the identification of transcriptional factors that regulate meiotically-active gene expression [46].

Since meiosis is a conserved process in all sexually reproducing eukaryotes, knowledge of gene function from one species could provide useful information transferable to other species. For example, studies in budding yeast (*Saccharomyces cerevisiae*) have revealed that a MER DNA helicase is required for the interference-sensitive pathway for crossover formation [67-71], and this finding led to the identification of a MER3 homolog, *ROCK-N-ROLLERS* (*RCK*) in *Arabidopsis*, supporting that as in budding yeast, both the interference-sensitive and insensitive pathways of recombination crossovers exist in plants [72,73]. Analysis of the “family history” of the meiotically-active genes from our study found a wide distribution of homologous sequences in many species in green plants (Viridiplantae), especially in flowering plants (Figure 5). This result suggests a great prospect of transferring the information obtained from *Arabidopsis* into other plants, including important crops such as soybean, maize, rice and Sorghum. Since low copies of putative homologous genes of *AtDMC1*, AT4G40020 and AT2G21640 seem to exist (Figure 5), exploring their correspondent promoter sequences in other species should be quite straightforward. For AT2G28090, *MS5*, AT1G26510, AT1G15320 and AT1G64625, many homologous genes were found in other plants and which might make it more difficult to elucidate “true” homologs and use their promoters; a more appropriate strategy in this case is to try to extend the usage of the promoter sequences from *Arabidopsis* directly to other plants.

In conclusion, we report here a bulk identification and experimental verification of meiotically-active promoters. The information provides not only invaluable clues about the meiotic regulatory system, but also a potential tool for the application in model and crop plants. In future work, it will be interesting and important to explore the relative activity levels of each promoter.

## Conclusions

In conclusion, the ten isolated promoter sequences were confirmed to specifically drive gene expression in meiocytes. The findings can provide important tools for meiosis studies and crop breeding, especially two of these promoters are prime candidates for meiosis directed-studies that desire an expression focused on early meiosis-I and/or meiosis-II.

## Methods

### Plant material and growth conditions

*Arabidopsis thaliana* (L.) Heynh. Ecotype *Columbia* (Col-0) was used in this study. Seeds were sown on 50% Sun Gro Professional Growing Mix and 50% Sun Gro SPECIAL BLEND growing medium (Sun Gro Horticulture, USA) and imbibed at 4°C for 3 days in the dark before moving them to long-day conditions (16 h light/8 h dark) at 22°C, 40 to 60% RH and 63 mE`·s^-1^·m^-2^ light intensity. The ER marker line ER-gk was obtained from the *Arabidopsis* Biological Resource Center (ABRC) [74].

### Examination of GFP fluorescence

Young inflorescences were dissected with syringe needles and the anthers were collected using 1xPBS buffer. The meiocytes were squashed out between a microscope slide and a cover glass. The GFP fluorescence was observed under an ERNST LEITZ WETZLAR 307–143.004 microscope (Wetzlar, Germany) and photographed with a SPOT Insight 4 Camera (Diagnostic Instruments, USA).

### Cloning, vector construction and plant transformation

To clone the promoters, the genomic sequences upstream of the candidate genes’ start codons were amplified using specific primers as listed in Table 1, in which *Sal*I or *Pst*I restriction sites were introduced for sense primers and *Nco*I sites for antisense primers. In order to prevent overlap with neighbouring genes or to get the appropriate primer binding sites, the cloned regions ranged from 0.6 Kb to 1.8 Kb, covering the key *cis*-elements of most promoters [75,76]. The *DMC1* promoter, which has a length of 3.0 Kb, is an exception, since it was cloned in our previous unpublished study. The promoters were cloned into the pCAMBIA1302 expression vector that contains the *mgfp5* version of the *Aequoria victoria* GFP [77], substituting the CaMV 35 S promoter upstream of the GFP coding sequence. The sequences were confirmed and the plasmids were introduced into *Agrobacterium tumefaciens* C58. To test the constructs *in planta*, all plasmids were introduced to the *Arabidopsis* plants using a floral dip method [78]. Transgenic plants were first screened on medium containing 40 mg/l hygromycin and transferred to soil, and further validated by PCR with the sense primers used in the promoter cloning (Table 1) and an antisense primer near the 5’ end of GFP (GTT GCA TCA CCT TCA CCC TCT).

### Analysis of *cis*-regulatory promoter elements

Known CREs were found by analysis with the PLACE database (http://www.dna.affrc.go.jp/PLACE/) [79-81]. The Pscan program was used to search for significantly overrepresented elements http://159.149.109.9/pscan/, [47]]. 1000 bp regions with respect to the annotated transcription start site of promoters were analyzed. The frequency matrices and visual logos of the sequences were obtained from the JASPAR CORE database. The *p*-values were computed by Pscan after *z*-test. An element was considered to be significantly overrepresented if the *p*-value was less than 0.1.

## Competing interests

The authors declare that they have no competing interests.

## Authors’ contributions

C.C. and E.F.R. conceived the project, designed the experiments and helped to draft the manuscript. J.L. carried out the molecular genetic studies, *cis*-element studies and drafted the manuscript. J.L. and C.C. performed the GFP reporter assay. A.D.F, I.E.L. and J.M. performed the homologs study. S.D. performed the endoplasmic reticulum marker assay and helped to draft the manuscript. T.L. helped in plant transformation and plant care. All authors read and approved the final manuscript.

## Supplementary Material

Additional file 1 Figure S1.GFP signals in meiocytes driven by meiotically-active promoters. (PDF 2536 kb). Click here for file

Additional file 2 Figure S2.The position of GFP spots and nuclear DNA/chromosomes in pMS5: GFP transformants. (PDF 636 kb). Click here for file

Additional file 3 Figure S3.GFP signals in both somatic and meiotic cells driven by a constitutive promoter d35S. (PDF 388 kb). Click here for file

Additional file 4 Table S1.PLACE motifs enriched in promoters of meiotically-active genes. (PDF 12 kb). Click here for file

Additional file 5 Table S2.Statistically overrepresented motifs in the promoters of meiotically-active genes. (PDF 8 kb). Click here for file

Additional file 6 Figure S4.Sequence logos of novel motifs in meiotically-active promoters found by MEME. (PDF 213 kb). Click here for file

Additional file 7 Table S3.A list of homologous recombination-related genes for comparative promoter analysis. (PDF 7 kb). Click here for file

Additional file 8 Figure S5.Sequence logos of novel motifs in promoters of homologous recombination-related genes found by MEME. (PDF 68 kb). Click here for file

Additional file 9 Table S4.Motifs enriched specifically in 15 known meiosis-related promoters. (PDF 7 kb) Click here for file
