# Supplementary material for: Characterization of a set of novel meiotically-active promoters in Arabidopsis
Source: BMC Plant Biol. 2012 Jul 9;12:104. doi: 10.1186/1471-2229-12-104 (PMC3462685; doi:10.1186/1471-2229-12-104)
Supplement: Additional file 1 — Figure S1.GFP signals in meiocytes driven by meiotically-active promoters. (PDF 2536 kb). [file 1471-2229-12-104-S1.pdf]

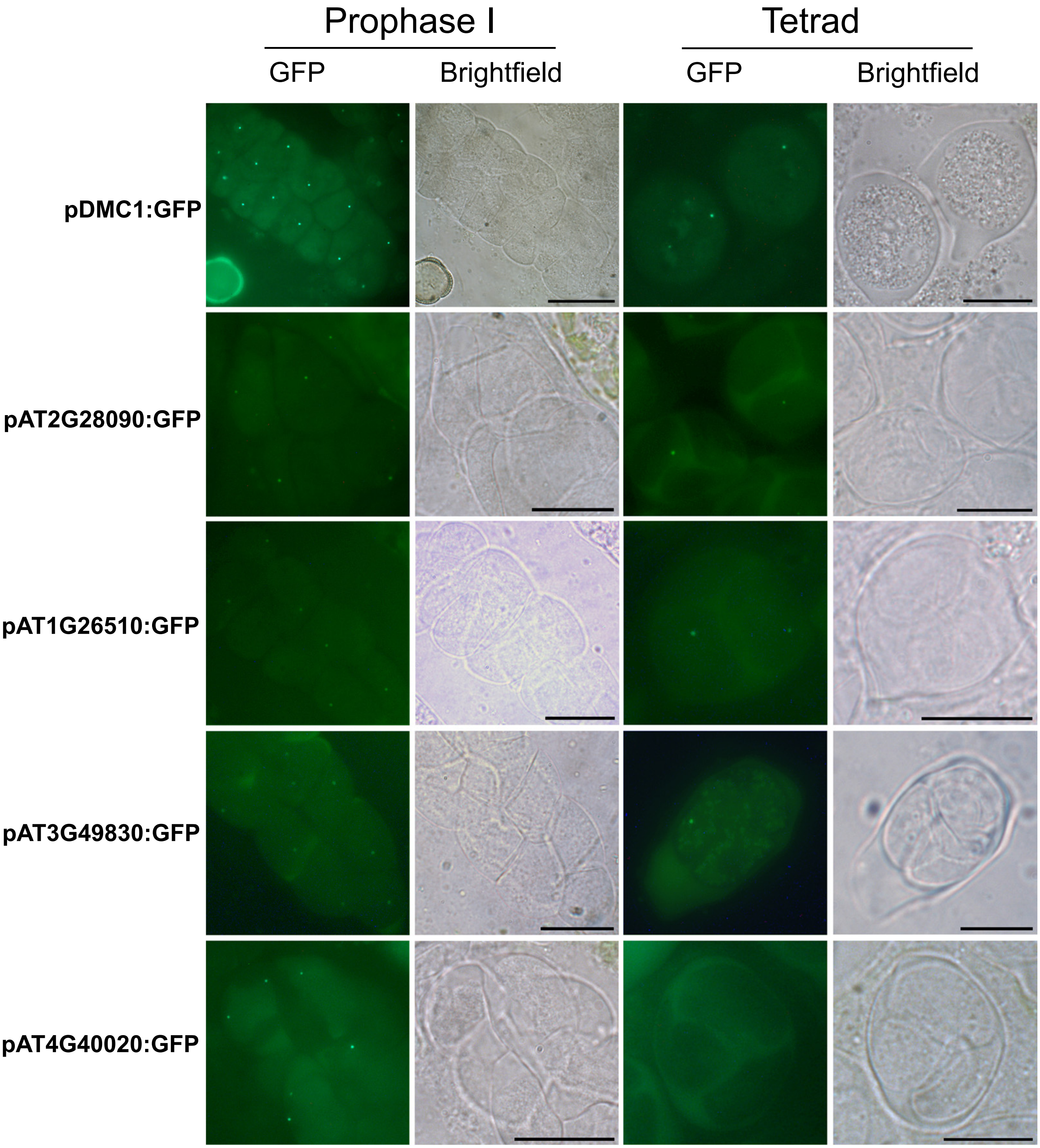


**Figure S1** GFP signals in meiocytes driven by meiotic active promoters. The name of the respective construct is on the left. Each GFP image has a corresponding bright-field image on the right. Scale bars, 10 μm.

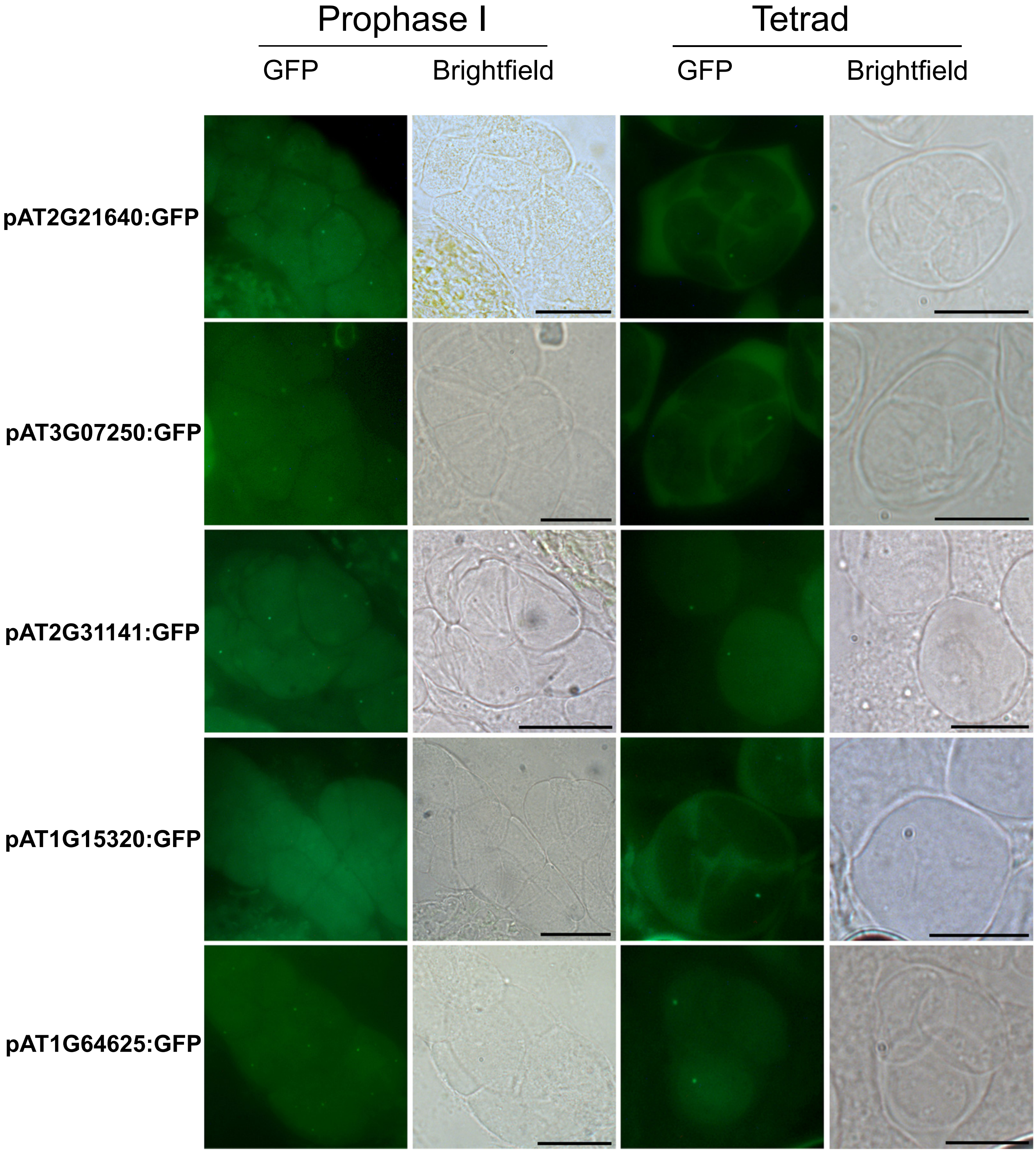


Figure S1 (continued)
