# Supplementary material for: Characterization of a set of novel meiotically-active promoters in Arabidopsis
Source: BMC Plant Biol. 2012 Jul 9;12:104. doi: 10.1186/1471-2229-12-104 (PMC3462685; doi:10.1186/1471-2229-12-104)
Supplement: Additional file 2 — Figure S2.The position of GFP spots and nuclear DNA/chromosomes in pMS5: GFP transformants. (PDF 636 kb). [file 1471-2229-12-104-S2.pdf]

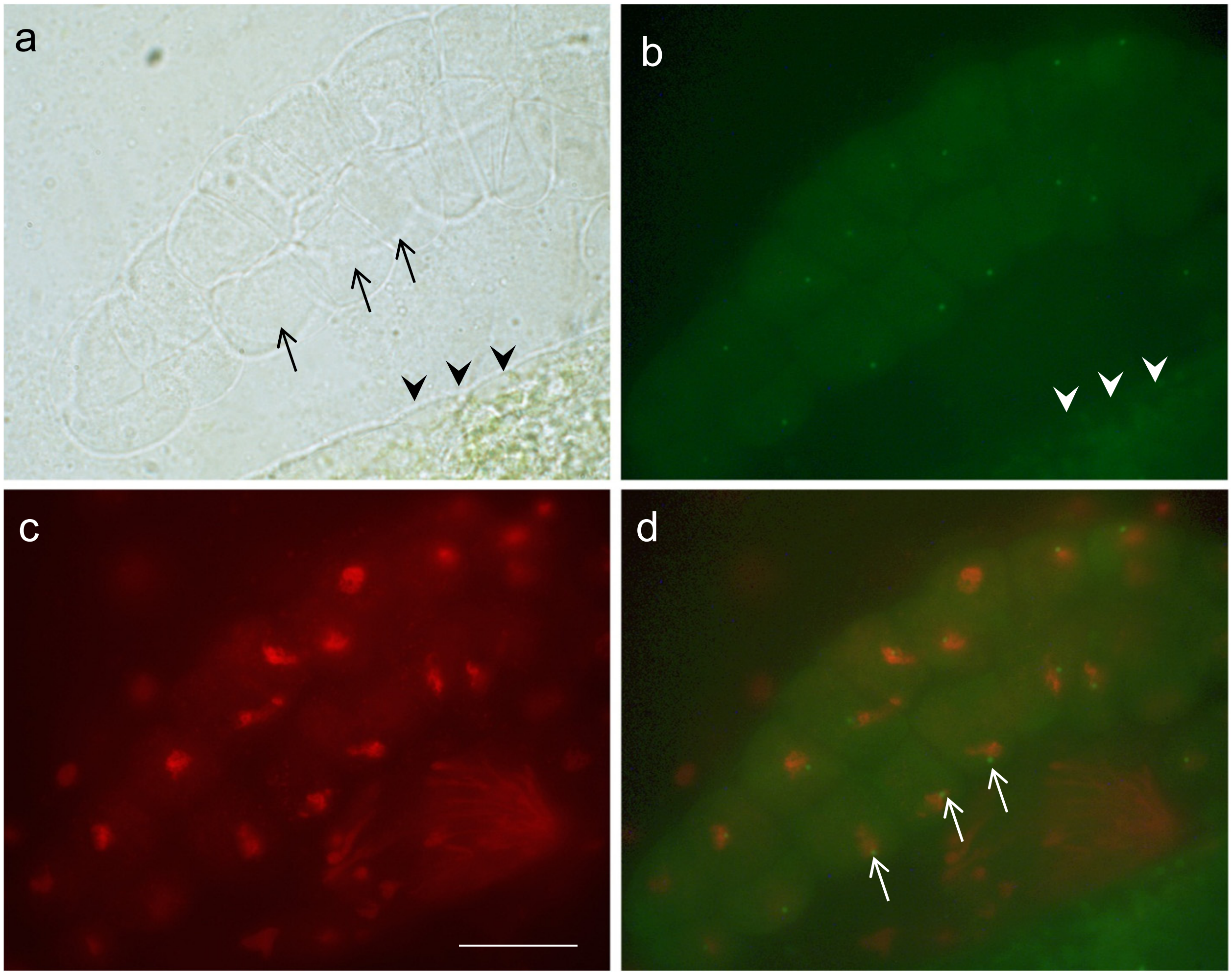

Figure S2 The position of GFP spots and nuclear DNA/chromosomes in pMS5: GFP transformants. (a), Brightfield, showing a male meiocyte column (black arrows) and somatic cells (black arrow heads); (b), observation of the GFP signals in pMS5:GFP fusion, showing no GFP signal detected in somatic cells (white arrowheads).; (c), artificially colored DAPI stained nuclear DNA/chromosomes (red); (d) a merge of (b) and (c), showing GFP spots are localized at the perinuclear region, each cell only bears one GFP spot (white arrows). Scale bar, 10um.
