# Supplementary material for: Characterization of a set of novel meiotically-active promoters in Arabidopsis
Source: BMC Plant Biol. 2012 Jul 9;12:104. doi: 10.1186/1471-2229-12-104 (PMC3462685; doi:10.1186/1471-2229-12-104)
Supplement: Additional file 3 — Figure S3.GFP signals in both somatic and meiotic cells driven by a constitutive promoter d35S. (PDF 388 kb). [file 1471-2229-12-104-S3.pdf]

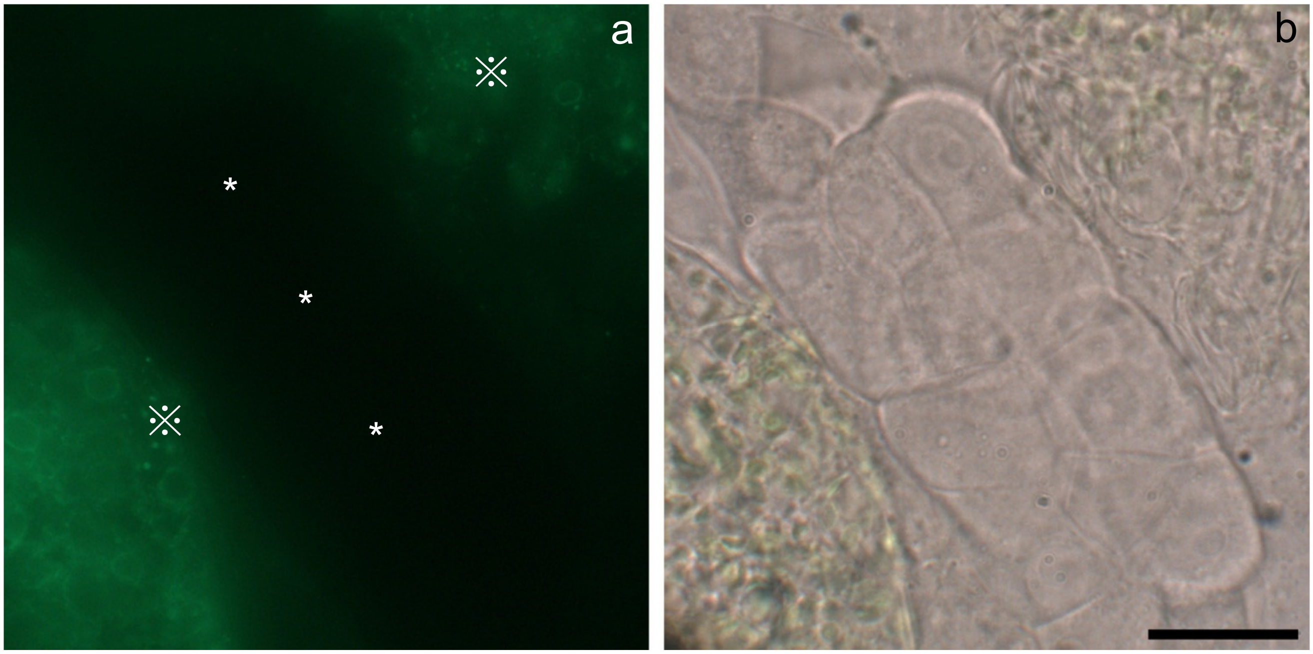

Figure S3 GFP signals in both somatic and meiotic cells driven by a constitutive promoter. The GFP signals from an endoplasmic reticulum marker are driven by a CaMV 35S promoter with dual enhancer elements (d35S). (a) shows GFP signals only in somatic cells (※), not in the meiosis I cluster (*); (b) is the corresponding bright-field image. Scale bars, 10 μm
