# Supplementary material for: Characterization of a set of novel meiotically-active promoters in Arabidopsis
Source: BMC Plant Biol. 2012 Jul 9;12:104. doi: 10.1186/1471-2229-12-104 (PMC3462685; doi:10.1186/1471-2229-12-104)
Supplement: Additional file 4 — Table S1.PLACE motifs enriched in promoters of meiotically-active genes. (PDF 12 kb). [file 1471-2229-12-104-S4.pdf]

Table S1 PLACE motifs enriched in promoters of meiotically-active genes.

PLACE motif	Motif pattern	Brief description
DOFCOREZM *	AAAG	Core site for binding of Dof proteins.
ARR1AT*	NGATT	Binding element of a response regulator ARR1.
CACTFTPPCA1*	YACT	A key component of Mem1 that determines the mesophyll-specific expression of *ppcA1*.
CAATBOX1*	CAAT	A motif associated with a seed storage protein gene.
GT1CONSENSUS*	GRWAAW	A consensus binding site in many light-regulated genes.
POLLEN1LELAT52 *	AGAAA	One of two co-dependent regulatory elements responsible for the pollen specific activation of tomato *LAT52*.
GATABOX *	GATA	Required for high level, light regulated and tissue specific control of transcription.
EBOXBNNAPA	CANNTG	E-box of *napA* storage-protein gene.
MYCCONSENSUSAT	CANNTG	MYC recognition site found in the promoters of the dehydration-responsive gene *rd22* and many other genes in *Arabidopsis*; binding site of ICE1; also known as RRE (R response element).
GTGANTG10*	GTGA	Plays an important role in modulating the expression of tobacco late pollen gene *g10* and tomato gene *lat56*.
WRKY71OS*	TGAC	A core of TGAC-containing W-box, the W-box elements within *PR-10* genes are binding site of parsley WRKY protein; binding site of rice WRKY71, a transcriptional repressor of the gibberellin signaling pathway.
ACGTATERD1	ACGT	Required for etiolation-induced expression of *erd1* in *Arabidopsis*.
ROOTMOTIFTAPOX1	ATATT	Motif found both in promoters of *rolD*.
NODCON2GM *	CTCTT	A putative nodulin consensus sequence.
OSE2ROOTNODULE*	CTCTT	One of the consensus sequence motifs of organ-specific elements (OSE), characteristic of the promoters activated in infected cells of root nodules.
RAV1AAT*	CAACA	Binding consensus sequence of *Arabidopsis* transcription factor RAV1; expression levels of *RAV1* are relatively high in rosette

		leaves and roots.
TAAAGSTKST1	TAAAG	Found in promoter of *Solanum tuberosum KST1* gene; target site for trans-acting StDof1 protein controlling guard cell-specific gene expression.
SEF4MOTIFGM7S*	RTTTTTR	Consensus sequence found in 5' upstream region of beta-conglycinin (7S globulin) gene; binding site for SEF4.
WBOXNTERF3	TGACY	May be involved in activation of *ERF3* gene by wounding.
IBOXCORE	GATAA	Conserved sequence upstream of light-regulated genes.
MYBCORE	CNGTTR	Binding site for all animal MYB and two *Arabidopsis* MYB proteins (ATMYB1 and ATMYB2).
TATABOX5*	TTATTT	A functional TATA element.
WBOXATNPR1*	TTGAC	Found in promoter of *Arabidopsis NPR1* gene; a cluster of WRKY binding sites act as negative regulatory elements for the inducible expression of AtWRKY18.
GT1GMSCAM4	GAAAAA	Plays a role in pathogen- and salt-induced *SCaM-4* gene expression.
SURECOREATSULTR11*	GAGAC	Core of sulfur-responsive element found in the promoter of *SULTR1*.
CURECORECR	GTAC	The core of a copper-response element, involved in oxygen-response of these genes.
EECCRCAH1*	GANTTNC	Consensus motif of the two enhancer elements, EE-1 and EE-2; binding site of Myb transcription factor LCR1.
POLASIG1	AATAAA	Near upstream elements (NUE) in *Arabidopsis*.
POLASIG3	AATAAT	Consensus sequence for plant polyadenylation signal.
ABRELATERD1	ACGTG	ABRE-like sequence required for etiolation-induced expression of *erd1* in *Arabidopsis*.

N=G/A/C/T, Y=T/C, R=A/G, W=A/T ; * refers to motifs found in all meiotically-active promoters.
