# Supplementary material for: Characterization of a set of novel meiotically-active promoters in Arabidopsis
Source: BMC Plant Biol. 2012 Jul 9;12:104. doi: 10.1186/1471-2229-12-104 (PMC3462685; doi:10.1186/1471-2229-12-104)
Supplement: Additional file 5 — Table S2.Statistically overrepresented motifs in the promoters of meiotically-active genes. (PDF 8 kb). [file 1471-2229-12-104-S5.pdf]

Table S2 Statistically overrepresented motifs in the promoters of meiotically-active genes.

**MATRIX_ID**	***p*-value**	**Name/Class/Family of the TF**	**Description of the CRE**
MA0128.1	9.03657e-03	EMBP-1; Zipper-type; Leucine zipper	Involved in ABA-mediated stress-signaling pathway [52].
MA0044.1	1.52751e-02	HMG-1; Other alpha-helix; High mobility group	Binding site of the chromatin-associated protein HMG-1 [3, 48].
MA0045.1	4.32294e-02	HMG-I/Y; Other alpha-helix; High mobility group	Binding site of the chromatin-associated protein HMG-I/Y [48].
MA0129.1	4.67192e-02	TGA1A; Zipper-type; Leucine zipper	A positive light regulatory element [52].
MA0121.1	9.30809e-02	ARR10; Helix-turn-helix; Myb	Core binding site of motif B in ARR10 [50].
MA0096.1	9.96644e-02	bZIP910; Zipper-type; Leucine zipper	Binding site of two bZIP proteins that are expressed predominantly in vascular tissues, carpels and anthers in flowers [51].

TF = transcription factor.
