# Supplementary material for: Characterization of a set of novel meiotically-active promoters in Arabidopsis
Source: BMC Plant Biol. 2012 Jul 9;12:104. doi: 10.1186/1471-2229-12-104 (PMC3462685; doi:10.1186/1471-2229-12-104)
Supplement: Additional file 7 — Table S3.A list of homologous recombination-related genes for comparative promoter analysis. (PDF 7 kb). [file 1471-2229-12-104-S7.pdf]

Table S3 A list of homologous recombination-related genes for comparative promoter analysis.

Name	AGI Identifier
AtSPO11-1	AT3G13170
AtSPO11-2	AT1G63990
AtMRE11	AT5G54260
AtRAD50	AT2G31970
AtRAD51	AT5G20850
AtRAD51C	AT2G45280
AtXRCC3	AT5G57450
AtMSH4	AT4G17380
AtMSH5	AT3G20475
RCK (AtMER3)	AT3G27730
AtPTD	AT1G12790
AtMUS81	AT4G30870
AtMLH3	AT4G35520
SDS	AT1G14750
TAM	AT1G77390
**Total**	15
