# Supplementary material for: Characterization of a set of novel meiotically-active promoters in Arabidopsis
Source: BMC Plant Biol. 2012 Jul 9;12:104. doi: 10.1186/1471-2229-12-104 (PMC3462685; doi:10.1186/1471-2229-12-104)
Supplement: Additional file 9 — Table S4.Motifs enriched specifically in 15 known meiosis-related promoters. (PDF 7 kb) [file 1471-2229-12-104-S9.pdf]

Table S4 Motifs enriched specifically in 15 known meiosis-related promoters.

MATRIX_ID	***p*-value**	Name/Class/Family of the TF	Description of the CRE
MA0034.1	2.28025e-02	Gamyb; Helix-turn-helix; Myb	Binding site of GAMYB which is expressed in cereal aleurone cells in response to gibberellin [80].
MA0044.1	2.41054e-02	HMG-1; Other alpha-helix; High mobility group	Binding site of the chromatin-associated protein HMG-1 [3, 48].
MA0008.1	9.1623e-02	HAT5; Helix-turn-helix; Homeodomain	Binding site of HAT5, a transcription activator involved in leaf development [81].
