# Supplementary material for: Characterization of a set of novel meiotically-active promoters in Arabidopsis
Source: BMC Plant Biol. 2012 Jul 9;12:104. doi: 10.1186/1471-2229-12-104 (PMC3462685; doi:10.1186/1471-2229-12-104)
Supplement: Additional file 6 — Figure S4.Sequence logos of novel motifs in meiotically-active promoters found by MEME. (PDF 213 kb). [file 1471-2229-12-104-S6.pdf]

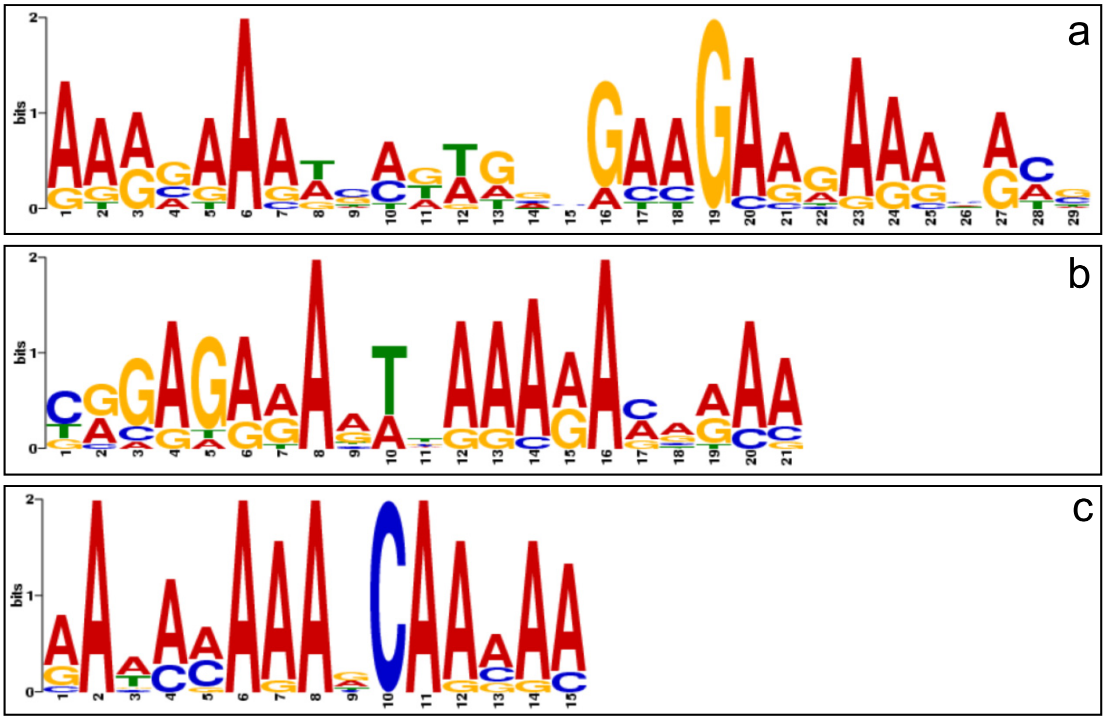

Figure S4 Sequence logos of novel motifs in meiotically-active promoters found by MEME. (a), E-value: 2.8e-001; found within 12 promoters. (b), E-value: 2.2e+002; found within 12 promoters. (c), E-value: 8.7e+001; found within 12 promoters.
