# Supplementary material for: Characterization of a set of novel meiotically-active promoters in Arabidopsis
Source: BMC Plant Biol. 2012 Jul 9;12:104. doi: 10.1186/1471-2229-12-104 (PMC3462685; doi:10.1186/1471-2229-12-104)
Supplement: Additional file 8 — Figure S5.Sequence logos of novel motifs in promoters of homologous recombination-related genes found by MEME. (PDF 68 kb). [file 1471-2229-12-104-S8.pdf]

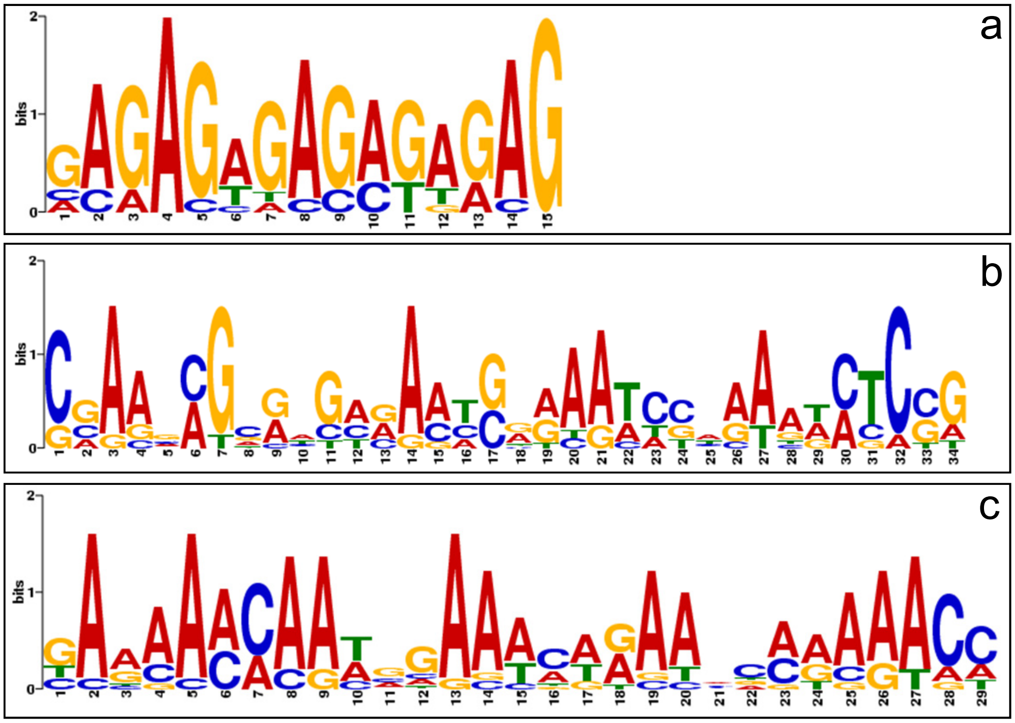

Figure S5 Sequence logos of novel motifs in promoters of homologous recombination-related genes found by MEME. (a), E-value: 7.2e-005; found within 11 promoters. (b), E-value: 2.9e+001; found within ten promoters. (c), E-value: 3.7e+001; found within 13 promoters.
